# Editorial: Insights in precision medicine: 2022

**DOI:** 10.3389/fmed.2023.1341175

**Published:** 2024-01-16

**Authors:** Alice Chen

**Affiliations:** AP Chen Consultant, Potomac, MD, United States

**Keywords:** artificial intelligence, prediction, genetics, cancer, modeling

As we step into the third decade of the 21st century, the scientific community has witnessed remarkable achievements, particularly in recent years, catapulting the field of Precision Medicine into unprecedented realms of advancement. Recognizing the need to stay abreast of these breakthroughs, the Research Topic “*Insights in precision medicine: 2022*” aim to showcase the developments in various scientific domains, emphasizing new insights, novel advancements, current challenges, latest discoveries, recent strides, and future perspectives within the expansive arena of Precision Medicine. The major theme of the articles published under this Research Topic focused on the use of artificial intelligence in medicine.

From the Oxford Dictionary of Phrase and Fable, artificial intelligence is defined as “the theory and development of computer systems able to perform tasks normally requiring human intelligence, such as visual perception, speech recognition, decision-making, and translation between languages”.^1^ Artificial intelligence (AI) has emerged as a pivotal force in the landscape of precision medicine and, indeed, the broader field of medicine. This is underscored by the corpus of articles featured in this Research Topic, collectively signaling a paradigm shift. Precision medicine, once hampered by lack of information now face the challenge of overwhelming volume of data that surpassed conventional processing capabilities, especially in the era of Next-Generation Sequencing (NGS). The pervasive integration of artificial intelligence (AI) in data processing has substantially enhanced our capacity to substantiate hypotheses, ushering in an era where inquiries that were once arduous and time-intensive now find resolution with newfound efficiency. The burgeoning capability to harness AI for analytical endeavors not only expedites the research process but also empowers scientists and researchers to address questions that were historically formidable and resource demanding. This transformative paradigm shift not only accelerates the pace of scientific discovery but also expands the horizons of what can be explored and understood, marking a notable evolution in the way we approach and unravel complex research challenges.

AI has demonstrated its capacity to sift through vast datasets, identifying intricate patterns, be it mutations within specific histologies or spanning across multiple histologies in an agnostic manner. This capacity opens new frontiers for uncovering potential therapeutic targets and agents. The compendium of articles encapsulated in the “Insights into Precision Medicine 2022” Research Topic elucidates the multifaceted applications of AI in the realm of medicine. Each article delineates unique use cases of AI, propelling the field forward by either contributing positive predictive factors or elucidating non-significant ones. Importantly, manuscripts revealing a lack of predictive value are underscored as being equally valuable as their positive counterparts. Encompassing a broad spectrum of subjects, this Research Topic delves into an array of medical domains, ranging from Idiopathic Pulmonary Fibrosis to nodular thyroid disease and diverse oncological conditions. Notably, it extends its purview to occupational hazards, exemplified in Zhao et al.'s manuscript, “*Predicting the risk of nodular thyroid disease in coal miners based on different machine learning models*.” These contributions represent pioneering forays into the integration of machine learning into the realm of medicine, marking a transformative shift.

The landscape, while promising, is not devoid of challenges. Concerns about the precision of data, the intricacies of data entry, and the nuances of interpretation loom large. Yet, these challenges are inherent in nascent stages, and their resolution is an ongoing process, poised to evolve as subsequent endeavors build upon the foundation laid by early adopters. The trajectory of this exploration into machine learning in medicine holds the promise of refining accuracy and efficacy, thus fostering a future where these concerns are progressively mitigated.

## Author contributions

AC: Writing—original draft, Writing—review & editing.

## Footnotes

^1^ https://www.oxfordreference.com/display/10.1093/oi/authority.20110803095426960#:~:text=The%20theory%20and%20development%20of,Dictionary%20of%20Phrase%20and%20Fable%20%C2%BB

